# Construction of a pancreatic cancer prediction model for oxidative stress-related lncRNA

**DOI:** 10.1007/s10142-023-01048-6

**Published:** 2023-04-05

**Authors:** Hao Huang, Yaqing Wei, Hao Yao, Ming Chen, Jinjin Sun

**Affiliations:** Department of Hepatopancreatobiliary Surgery, The Second Hospital of Tianjin Medical University, Tianjin, China

**Keywords:** Pancreatic cancer, Oxidative stress-related lncRNA, Biomarker, Prognostic model, TCGA-PAAD, ICGC-PACA

## Abstract

Long non-coding RNAs (lncRNAs) may play a role in oxidative stress by altering the tumor microenvironment, thereby affecting pancreatic cancer progression. There is currently limited information on oxidative stress-related lncRNAs as novel prognostic markers of pancreatic cancer. Gene expression and clinical data of patients with pancreatic cancer were downloaded from The Cancer Genome Atlas (TCGA-PAAD) and the International Cancer Genome Consortium (ICGC-PACA) database. A weighted gene co-expression network analysis (WGCNA) was constructed to identify genes that were differentially expressed between normal and tumor samples. Based on the TCGA-PAAD cohort, a prediction model was established using lasso regression and Cox regression. The TCGA-PAAD and ICGC-PACA cohorts were used for internal and external validation, respectively. Furthermore, a nomogram based on clinical characteristics was used to predict mortality of patients. Differences in mutational status and tumor-infiltrating immune cells between risk subgroups were also explored and model-based lncRNAs were analyzed for potential immune-related therapeutic drugs. A prediction model for 6-lncRNA was established using lasso regression and Cox regression. Kaplan–Meier survival curves and receiver operating characteristic (ROC) curves indicated that patients with lower risk scores had a better prognosis. Combined with Cox regression analysis of clinical features, risk score was an independent factor predicting overall survival of patients with pancreatic cancer in both the TCGA-PAAD and ICGC-PACA cohorts. Mutation status and immune-related analysis indicated that the high-risk group had a significantly higher gene mutation rate and a higher possibility of immune escape, respectively. Furthermore, the model genes showed a strong correlation with immune-related therapeutic drugs. A pancreatic cancer prediction model based on oxidative stress-related lncRNA was established, which may be used as a biomarker related to the prognosis of pancreatic cancer to evaluate the prognosis of pancreatic cancer patients.

**Supplementary Information:**

The online version contains supplementary material available at 10.1007/s10142-023-01048-6.

## Introduction

Pancreatic cancer is one of the most malignant tumors in the world. It currently accounts for about 7% of all cancer-related deaths worldwide, ranking third after colon cancer and lung cancer, and is prognosticated to occupy the second position by 2030 (Rahib et al., 2014). The most common type of pancreatic cancer is pancreatic ductal adenocarcinoma, which accounts for approximately 85% of all pancreatic cancers (Rawla et al., 2019). Due to the absence of early symptoms and effective methods of detection and treatment, the incidence rate of pancreatic cancer is practically comparable to that of mortality (Sung et al., 2021). Furthermore, there is insufficient evidence concerning risk factors for the development of pancreatic cancer and those that are recognized do not adequately explain the development of this malignancy; currently, risk factors are only identified in approximately 40% of cases (Capasso et al., 2018). Among them, the predominant environmental risk factors for pancreatic cancer are smoking, alcohol consumption, chronic pancreatitis, age, obesity, and diabetes.

Oxidative stress is usually caused by an imbalance between the production of reactive oxygen species (ROS) and the cellular antioxidant defense system. Oxidative stress is suggested to play a key role in the pathogenesis of pancreatitis, which is in turn an important risk factor for the development of pancreatic cancer (Swentek et al., 2021). In an inflammatory environment, abnormal pancreatic enzyme secretion and increased inflammatory responses can stimulate ductal metaplasia, which is a major cause of pancreatic precancerous lesions. Additionally, oxidation of DNA and subsequent genetic mutation, cell membrane breakdown, and oxidative stress, which causes protein misfolding, can promote carcinogenesis (Cykowiak & Krajka-Kuźniak, 2021). Furthermore, some studies have indicated that genetic changes that increase ROS production can promote cancer progression, while treatment with antioxidants can suppress metastasis (LeBleu et al., 2014). For example, inhibition of TIGAR, an enzyme that promotes the entry of glucose into the pentose phosphate pathway, increases ROS levels in pancreatic duct adenocarcinoma, resulting in increased migration, invasion, and metastasis (Cheung et al., 2020). In addition, oxidative stress can induce changes in the microenvironment, leading to the production and accumulation of potent tumor-stimulating components in the extracellular matrix (ECM) to advance cancer cell progression (Kim et al., 2022).

Oxidative stress is a feature of carcinogenesis, and excessive accumulation of ROS to promote tumorigenesis and progression requires aberrant redox homeostasis. The establishment of homeostasis is closely related to lncRNA. LncRNAs have been widely identified as multiple regulators involved in several key redox-sensing pathways, such as NF-κB and Nrf2 signaling, and thus may be effective targets for cancer therapy (Bhattacharjee et al., 2020; Ren et al., 2020). In addition, lncRNAs have the characteristics of convenient storage, acquisition, and screening, and less invasive detection methods, which are beneficial for clinicians monitoring redox homeostasis, as well as providing certain advantages as cancer biomarkers (Wang et al., 2022). The current study utilized pancreatic cancer samples in the databases to construct an oxidative stress lncRNA model to explore the characteristics of the lncRNA in terms of mutation status and tumor-infiltrating immune cells, as well as its potential clinical application as a biomarker and therapeutic target.

## Materials and methods

### Data acquisition and integration

The purpose of this analysis is to predict patient survival time based on the genetic model. To exclude patients who died due to factors such as postoperative complications, this analysis excluded samples with missing overall survival and overall survival of less than 30 days. Transcriptome data of 165 tumor samples and 171 normal tissue samples were downloaded and integrated from the Genotype-Tissue Expression (GTEx) and The Cancer Genome Atlas (TCGA) databases, and clinical information for the samples was obtained from the TCGA database for the subsequent validation of clinical characteristics and prognostic value of genes. Likewise, the transcriptome data of 90 tumor samples in PACA-CA cohort and their clinical data were downloaded and integrated from the International Cancer Genome Consortium (ICGC) database as an external validation dataset for the prognostic assessment of model genes. The DESeq2 R package was used to perform differential expression analysis on the samples obtained from the GTEx and TCGA databases under the conditions of log2FC >1.0, FDR <0.05, and *P* <0.05, and 5901 genes that met the conditions were considered potential target genes.

### Data processing and weighted gene co-expression network analysis (WGCNA) construction

The potential target genes were intersected with the genes obtained from ICGC database and the intersecting genes were retained. Genes in the top 75% of median absolute deviations (*MAD* >0.01) were screened using the WGCNA R package to construct a scale-free network evaluation map. The Pearson correlation coefficient and weighted adjacency matrix of genes and clinical traits were established by the power function a_GC_=|c_GC_|β (where c_GC_ is the Pearson correlation between genes (G) and clinical trait (C), and a_GC_ is the adjacency between genes and clinical trait). Subsequently, a suitable soft-threshold parameter β was screened to highlight correlations and penalize weak correlations between genes. The connections were then transformed into a topological overlap matrix (TOM), based on TOM’s dissimilarity measure, and average linkage hierarchical clustering was performed with a minimum module size of 260 for genes. According to the clustering results, correlation coefficients of 0.40 and 0.05 were selected to calculate the dissimilarity of the module eigengenes, respectively. Finally, Gene Ontology (GO) and Kyoto Encyclopedia of Genes and Genomes (KEGG) analyses were performed on genes associated with clinical prognosis (correlation coefficient =0.40, *P* <0.05).

### Identification of oxidative stress-related lncRNAs

The potential target genes were rescreened using the limma R package and Strawberry Perl was employed to distinguish lncRNAs (log2FC >1.0, FDR <0.05, and *P* <0.05). Subject genes in the GENCARD website (https://www.genecards.org/) with “oxidative stress” as the keyword (relevance score >6.0, gifts score >15.0) were downloaded, and subsequently, the intersection of the potential target genes and the subject genes was taken as the target genes. Correlation analysis between lncRNAs and target genes was then conducted, and lncRNAs were screened as potential model lncRNAs under the conditions of Pearson’s correlation coefficient >0.4, *P* <0.001.

### Establishment and validation of the risk signature

Univariate Cox proportional hazard regression analysis was used to screen lncRNAs related to survival from potential model lncRNAs (*P* <0.05). Subsequently, lasso regression with 10-fold cross-validation, a *P*-value of 0.05, and a run of 1000 loops was performed. For each loop, 1000 random stimuli were set to prevent overfitting. The results of lasso regression were analyzed by multivariate Cox proportional hazards regression, and the final model lncRNAs were determined (*P* <0.05). The risk score was then calculated with the following formula:

$$\textrm{risk}\ \textrm{score}=\sum\limits_{\textrm{k}=1}^{\textrm{n}}\textrm{coef}\left({\textrm{lncRNA}}^{\textrm{k}}\right)\times \textrm{expr}\left({\textrm{lncRNA}}^{\textrm{k}}\right)$$

where coef(lncRNAn) was the short form of the coefficient of lncRNAs correlated with survival, and expr(lncRNAn) was the expression of lncRNAs. According to the median risk score, subgroups were established that included low- and high-risk groups. To evaluate the prognostic value of the model, the Strawberry Perl and caret R package was used to randomly divide 165 tumor samples in the TCGA database into a training cohort and a validation cohort with a ratio of 1:1. Cross-validation of clinical characteristics between cohorts indicated that the cohorts were independent from each other. In addition, 90 tumor samples from the ICGC database were divided into low- and high-risk subgroups as an external validation of the model.

### Assess model clinical characteristics

For internal validation, the risk scores, survival status, and survival analysis curves based on low- and high-risk subgroups were constructed for the training and validation cohorts, respectively. Temporal ROC curves of the model at 1, 2, and 3 years were then plotted in the training and validation cohorts. In addition, based on the entire cohort, clinical characteristics such as age, gender, tumor grade, and tumor stage were compared between low- and high-risk subgroups. Likewise, in external validation, the risk scores, survival status, and survival analysis curves were constructed for 165 samples in the entire cohort and 90 samples in the ICGC cohort, respectively. Subsequently, temporal ROC and clinical characteristic-dependent ROC curves were plotted. In addition, risk scores and clinical characteristics were assessed using Cox regression analysis (data loss in 4 of 165 tumor samples). Finally, a nomogram was constructed based on clinical prognosis.

### Mutation data analysis

Relevant variant data were downloaded and integrated from the TCGA database, and the data variant status was browsed using the maftools R package. The top 30 genes in the mutation data were browsed and selected and three waterfall plots were drawn—one for the total sample of the top 10 mutated genes, and two for the top 20 mutated genes based on low- and high-risk subgroups. Excluding hyper-mutations samples (tumor mutational burden (TMB) >10.0), differences in TMB in low- and high-risk subgroups were analyzed for association with risk scores. The samples were then divided into L-TMB and H-TMB groups (low and high TMB, respectively) according to the median TMB, and survival curves were constructed in relation to survival time. Subsequently, the low- and high-risk subgroups in the model were combined with the L-TMB and H-TMB groups to construct survival curves. In addition, GSEA software was used to analyze pathways of gene enrichment in low- and high-risk subgroups.

### Analysis of tumor-infiltrating immune cells

Tumor-infiltrating immune cells data of the TCGA cohort were obtained and integrated from the Timer2 database (http://timer.comp-genomics.org/) for analysis, and literature was searched to obtain immune subtypes of the samples. A survival curve related to survival time was then constructed based on median immune cell infiltration scores (*P* <0.05), and the differences in immune cell scores of the microenvironment between low- and high-risk subgroups were analyzed through the XCELL database (https://xcell.ucsf.edu/) data. Subsequently, correlation analysis between risk score and immune cell infiltration score was conducted using limma, ggplot2, and ggpubr R packages. In addition, tumor immune dysfunction and exclusion (TIDE) scores of the samples were obtained through the website (http://tide.dfci.harvard.edu/), and the differences in TIDE scores between low- and high-risk subgroups were analyzed.

### Model-related genes and potential drug target predictions

Based on low- and high-risk subgroups, principal component analysis (PCA) was performed through the limma and scatterplot3d R packages to view the sample distribution. Subsequently, a correlation analysis between differentially expressed genes and model lncRNAs was conducted, and differentially expressed genes that were related to at least 3-model lncRNAs were screened to generate a correlation heatmap (*P* <0.05). Next, a Sankey diagram of target genes and model lncRNAs was constructed to view their expression in the samples (|correlation coefficient >0.4|, *P* <0.001). The pRRophetic R package was then used to assess treatment response in low- and high-risk subgroups based on the half-maximal inhibitory concentration of the samples (*P* <0.05). In addition, information about genes and drug targets was obtained and integrated from the CellMiner website (https://discover.nci.nih.gov/cellminer/home.do), and correlation between genes and drug targets was calculated through the limma R package to predict the potential therapeutic effect of drugs (*P* <0.05).

## Results

### Data preprocessing and construction of WGCNA

**Fig. 1:**
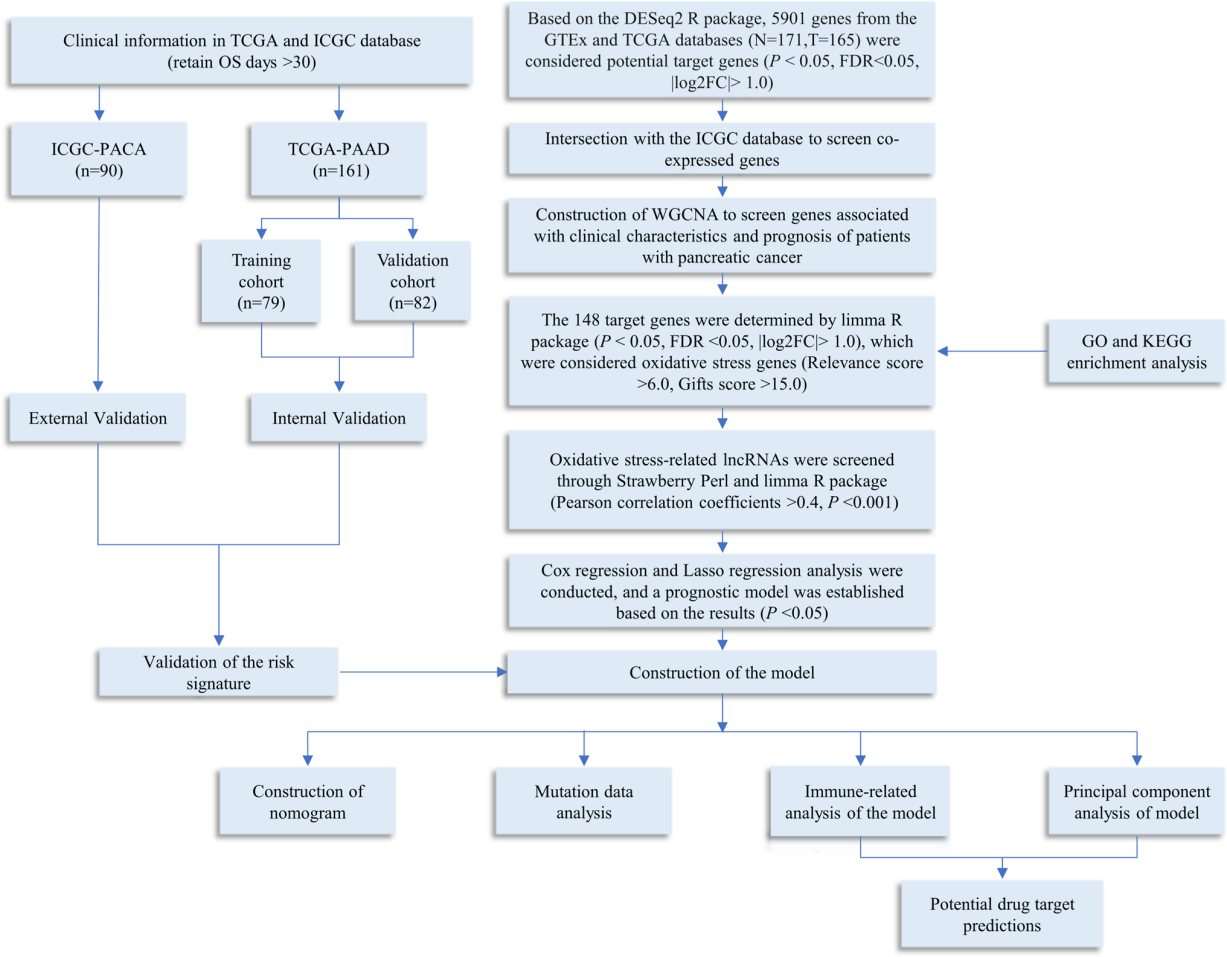
Flowchart of the study

**Fig. 2:**
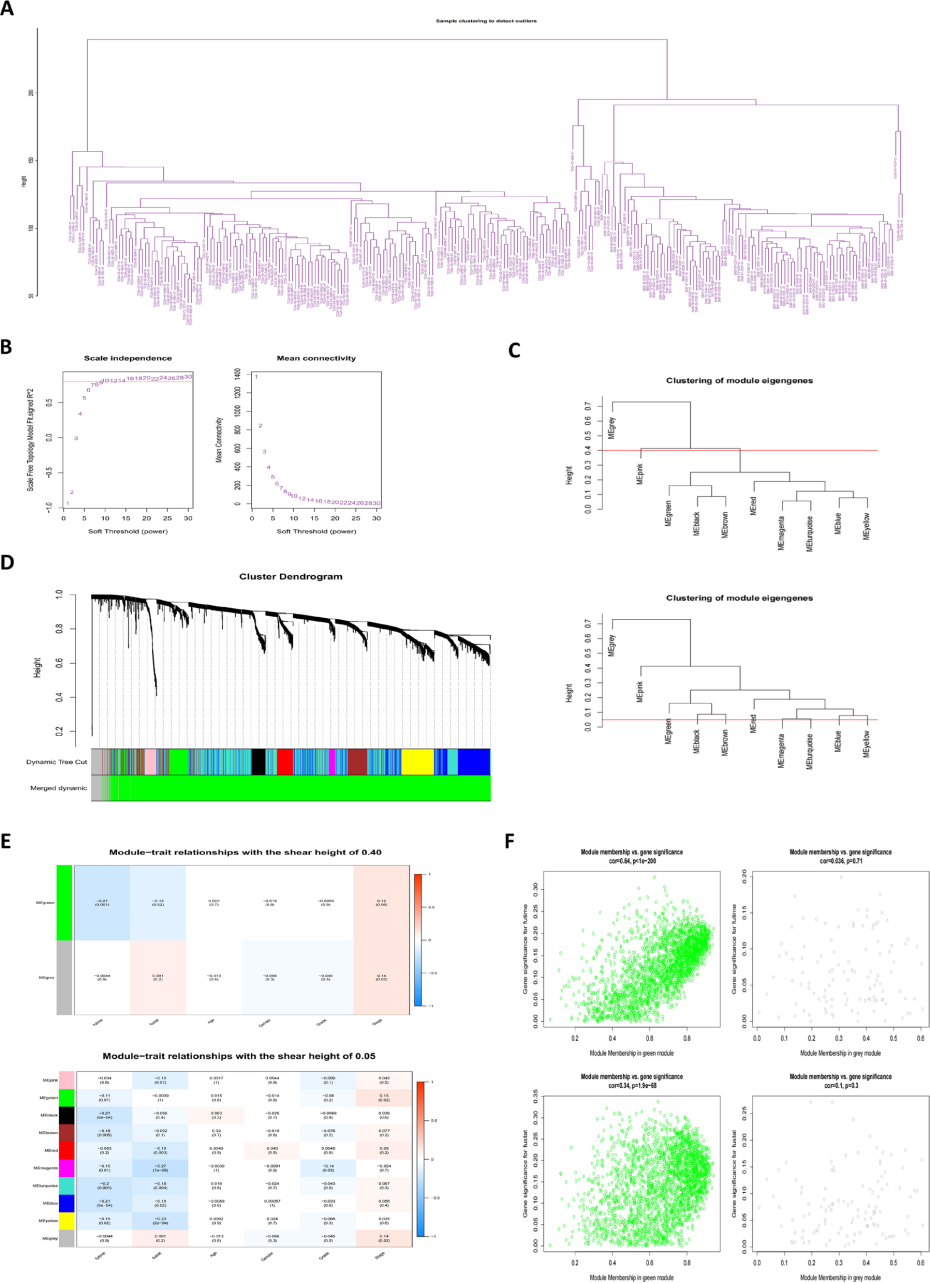
Construction of WGCNA. **A** Clustering of genes in samples from TCGA and ICGC databases to detect outliers. **B** The scale-free ft index for soft thresholding powers. **C** Clustering dendrogram of gene modules at shear heights of 0.40 and 0.05. **D** Dendrogram of the differentially expressed genes clustered on the basis of different metrics. **E** Heatmap showing the correlation between gene module and clinical traits. **F** Scatter plot of module eigengenes in green and grey modules with a shear height of 0.40

The research process is shown in Fig. 1. By analyzing 171 normal samples and 165 tumor samples in the GTEx and TCGA databases, 5901 differentially expressed genes were obtained. In addition, 3469 differentially co-expressed genes were obtained by intersecting the gene matrix of 90 samples in ICGC database (Supplementary Table S1). Subsequently, a WGCNA data network was constructed to filter out outlier samples (Fig. 2A; Supplementary Table S2) and the optimal soft threshold parameter *β*=10 was selected to construct the topological overlap matrix (Fig. 2B), taking the module correlation as 0.40 and 0.05 as a reference (Fig. 2C, D). Based on the topological overlap matrix data (Fig. 2E), the green module genes (Fig. 2F) that were related to both clinical traits and prognosis when the module correlation was 0.4 were retained for further analysis (*P* <0.05).

### GO and KEGG enrichment analysis

**Fig. 3:**
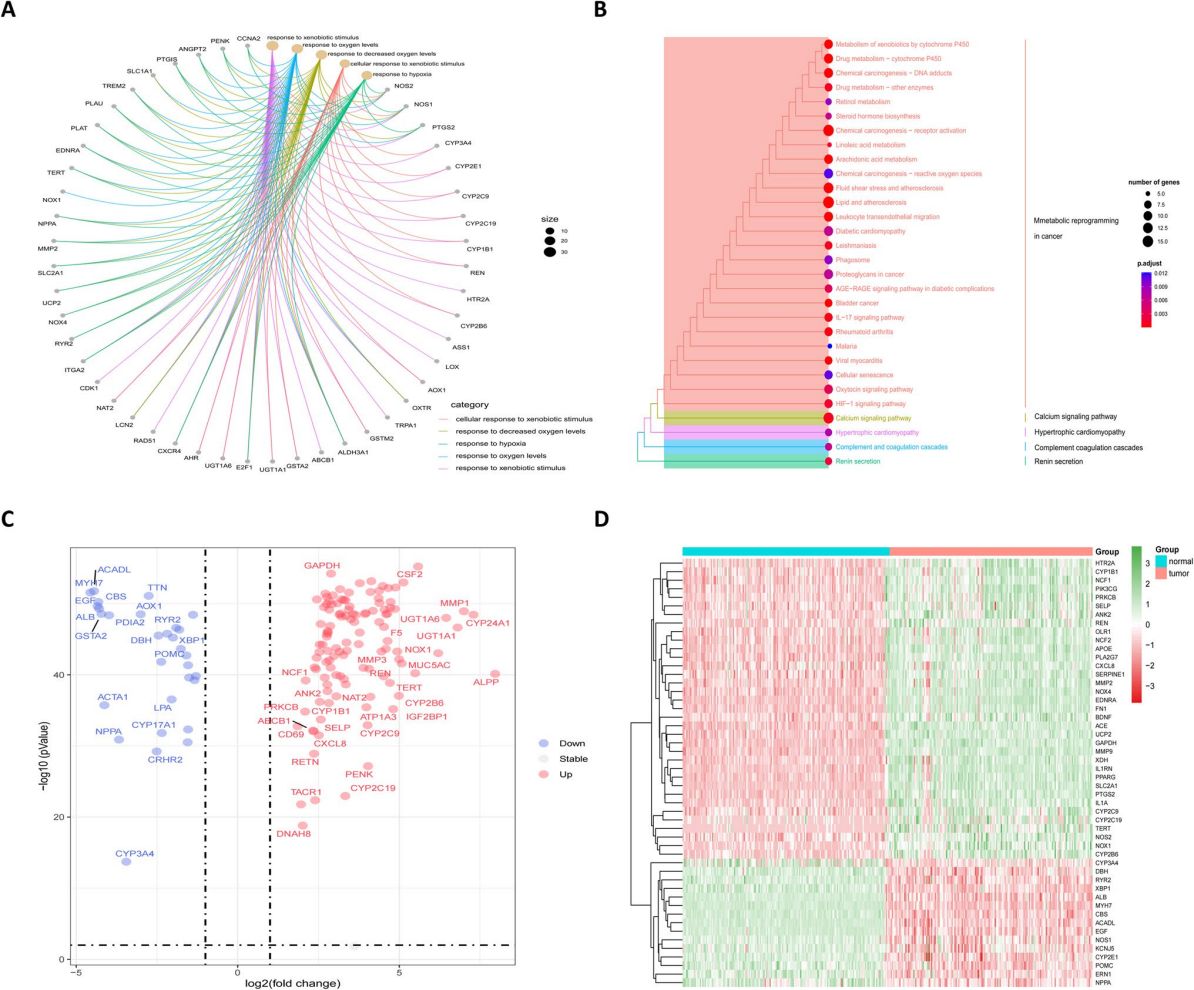
Functional enrichment analysis of differentially expressed genes. **A** Network diagram showing genes enriched in GO analysis. **B** Top 30 KEGG enrichment terms under the k-means clustering algorithm. **C** Volcano plot of 148 differentially expressed oxidative stress genes between 171 normal and 165 tumor samples in GTEx and TCGA databases. **D** Heatmap of top 50 differentially expressed oxidative stress genes

GO analysis was performed on the green module genes analyzed by WGCNA, and most genes were enriched in five pathways, including response to xenobiotic stimulus and response to oxygen levels (Fig. 3A). In the KEGG analysis, genes were enriched according to the k-means clustering algorithm, and most of the pathways were concentrated in “metabolic reprogramming in cancer” (Fig. 3B). In addition, a total of 148 target genes were plotted in a volcano plot (Fig. 3C; Supplementary Table S3), and genes with a log2 fold change value greater than 3 were annotated, including genes such as *GAPDH* and *REN*. Then, based on the *P*-value, the top 50 genes with significant differences were selected to draw a heatmap (Fig. 3D).

### Model construction and internal validation

**Fig. 4:**
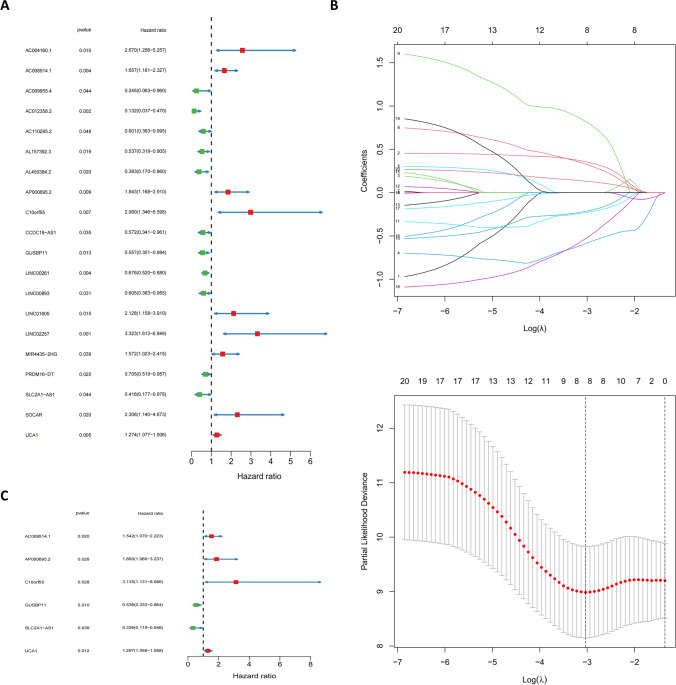
Cox regression and lasso regression analysis to determine model genes. **A** Univariate Cox regression analysis for identification of prognosis-associated oxidative stress lncRNAs. **B** Lasso regression analysis to reduce the number of factors to prevent overfitting. **C** Multivariate Cox regression analysis established a 6-lncRNAs prognostic model

**Fig. 5:**
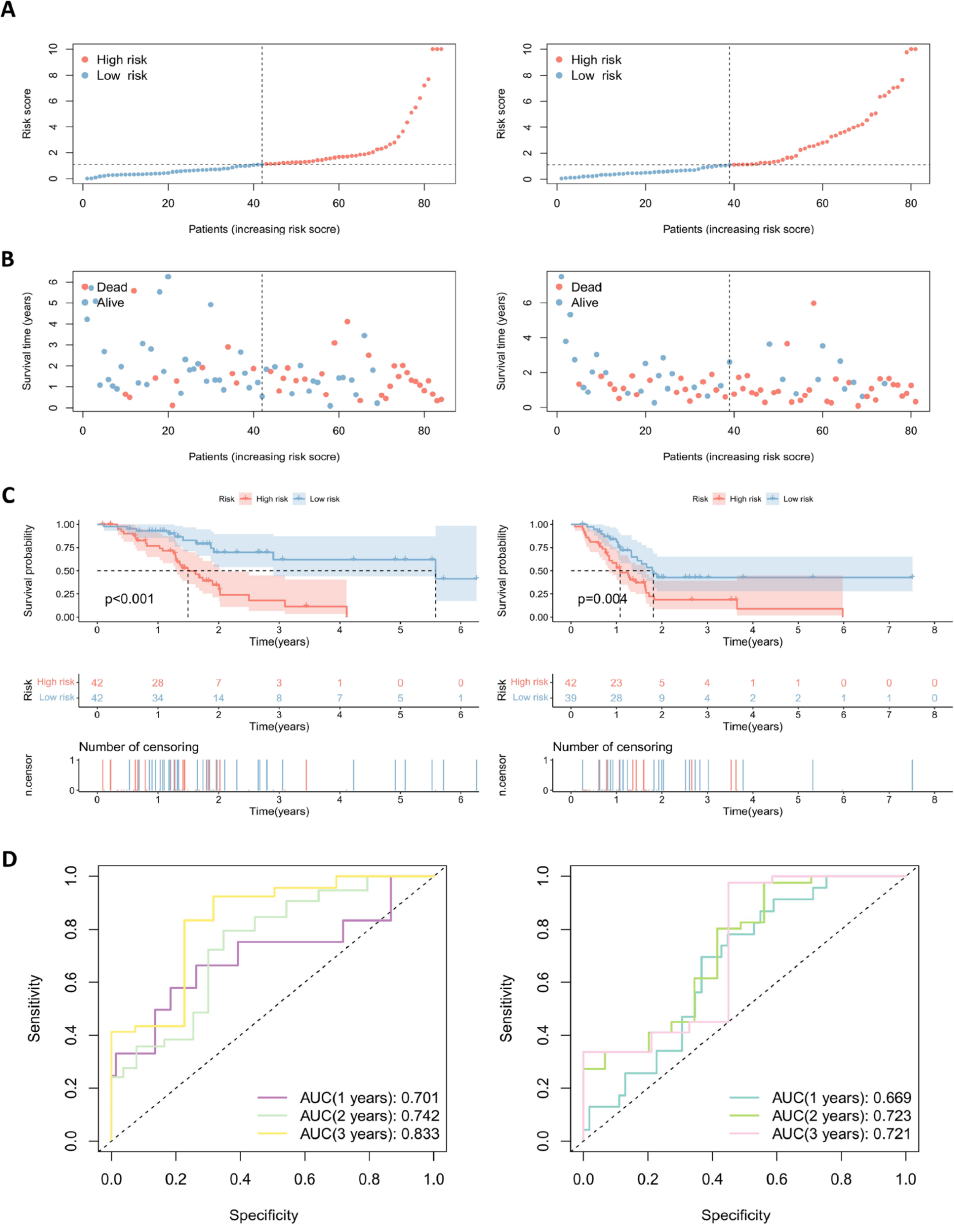
Internal validation of the prognostic model. **A** and **B** Distribution of risk score (**A**) and survival status (**B**) for risk subgroups in training (left) and validation (right) cohorts. **C** Kaplan–Meier survival curves for overall survival of patients in training (left) and validation (right) cohorts. **D** Temporal ROC curves for predicting 1-, 2-, and 3-year overall survival for patients in training (left) and validation (right) cohorts

**Fig. 6:**
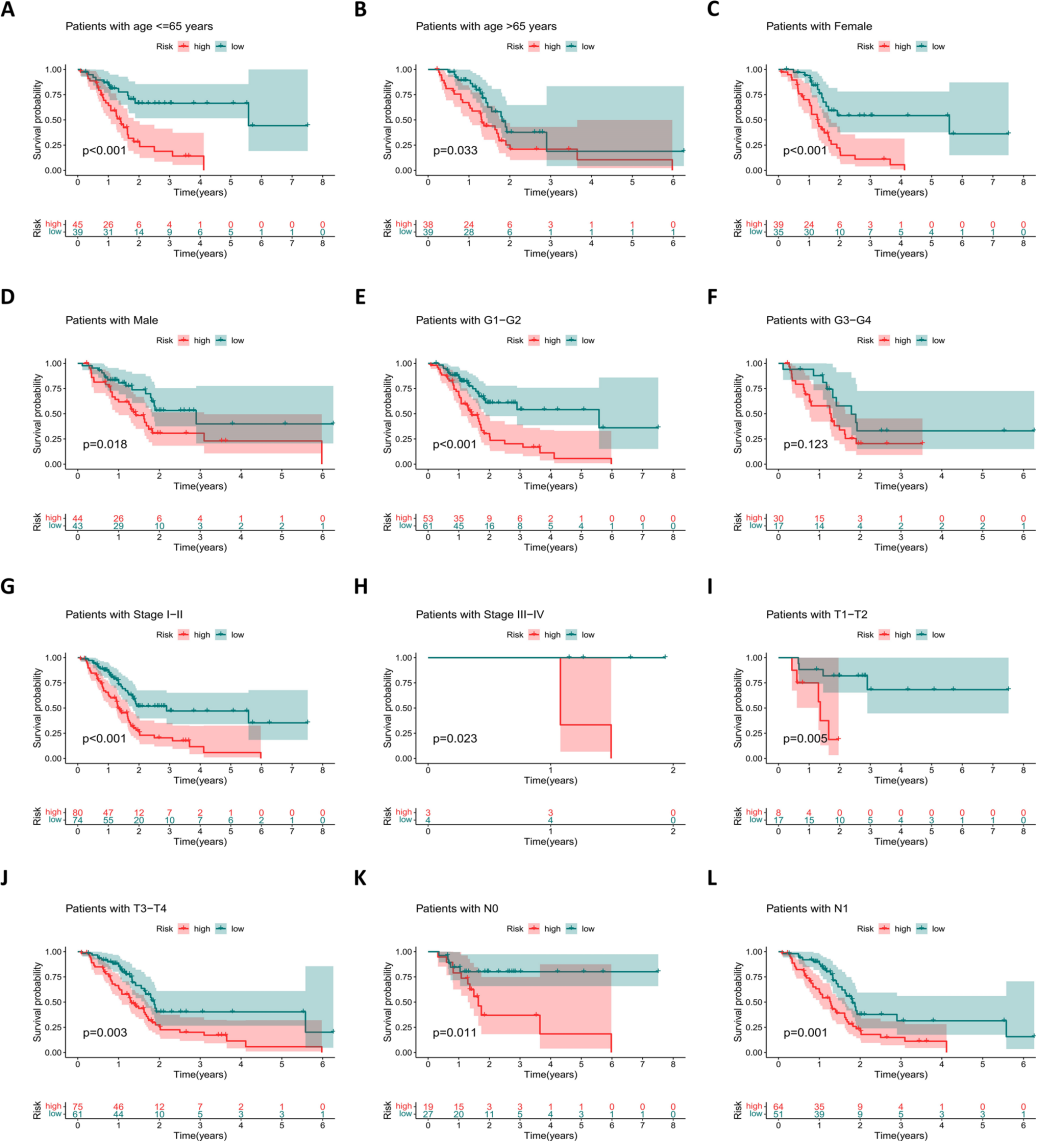
The overall survival prognostic values of Kaplan–Meier survival curves were stratified between low- and high-risk groups in the TCGA cohort by age (**A** and **B**), gender (**C** and **D**), tumor grade (**E** and **F**), stage of cancer (**G** and **H**), T-stage (**I** and **J**), and N-stage (**K** and **L**)

Univariate Cox regression analysis (Fig. 4A) was performed on the screened lncRNAs, and 20 lncRNAs associated with prognosis (*P* <0.05) were identified. Subsequently, lasso regression analysis (Fig. 4B) was performed to screen for eight prognosis-related lncRNAs when the first-order value of Log(λ) was the least likelihood of bias (*P* <0.05). Based on the results of multivariate Cox regression analysis (Fig. 4C), we established a prognostic model consisting of six lncRNAs: AC008514.1, AP000695.2, C10orf5, GUSBP11, SLC2A1-AS1, and UCA1. Among them, GUSBP11 and SLC2A1-AS1 exhibit protective effects in the model, and their high expression is beneficial to the prognosis of patients; However, AC008514.1, AP000695.2, C10orf5, and UCA1 are risk genes, and their high expression is unfavorable for the prognosis of patients (*P* <0.05). Subsequently, the prognostic scores calculated based on the expression of genes in the model grouped patients for internal validation (Table 1), and the low-risk groups in both the training and validation cohorts achieved better prognosis compared with the high-risk groups (Fig. 5A–C). In addition, the area under the ROC curve for both the training cohort and the validation cohort for 1 to 3 years was more than 0.66, which is relatively respectable (Fig. 5D). Finally, the validation of models for each clinical feature showed prognostic differences, which further confirmed the effectiveness of model grouping (Fig. 6A–L).

**Table 1:** Internally validated clinical information for the TCGA cohort

Clinical feature	Type	Training cohort	Validation cohort	Entire cohort	*P* value
Age (years)	≤65	43 (52.44%)	41 (51.90%)	84 (52.17%)	1.0000
	>65	39 (47.56%)	38 (48.10%)	77 (47.83%)	
Gender	Female	43 (52.44%)	31 (39.24%)	74 (45.96%)	0.1281
	Male	39 (47.56%)	48 (60.76%)	87 (54.04%)	
Tumor grade	G1	12 (14.63%)	13 (16.46%)	25 (15.53%)	0.4794
	G2	43 (52.44%)	46 (58.23%)	89 (55.28%)	
	G3	27 (32.93%)	19 (24.05%)	46 (28.57%)	
	G4	0 (0%)	1 (1.27%)	1 (0.62%)	
Tumor stage	I	8 (9.76%)	9(11.39%)	17 (10.56%)	0.9398
	II	70 (85.37%)	67(84.81%)	137 (85.09%)	
	III	2 (2.44%)	1(1.27%)	3 (1.86%)	
	IV	2 (2.44%)	2(2.53%)	4 (2.48%)	
T Stage	T1	1 (1.22%)	5 (6.33%)	6 (3.73%)	0.2339
	T2	12 (14.63%)	7 (8.86%)	19 (11.80%)	
	T3	67 (81.71%)	66 (83.54%)	133 (82.61%)	
	T4	2 (2.44%)	1 (1.27%)	3 (1.86%)	
N Stage	N0	25 (30.49%)	21 (26.58%)	46 (28.57%)	0.7085
	N1	57 (69.51%)	58 (73.42%)	115 (71.43%)

### External validation of model and construction of nomogram

**Fig. 7:**
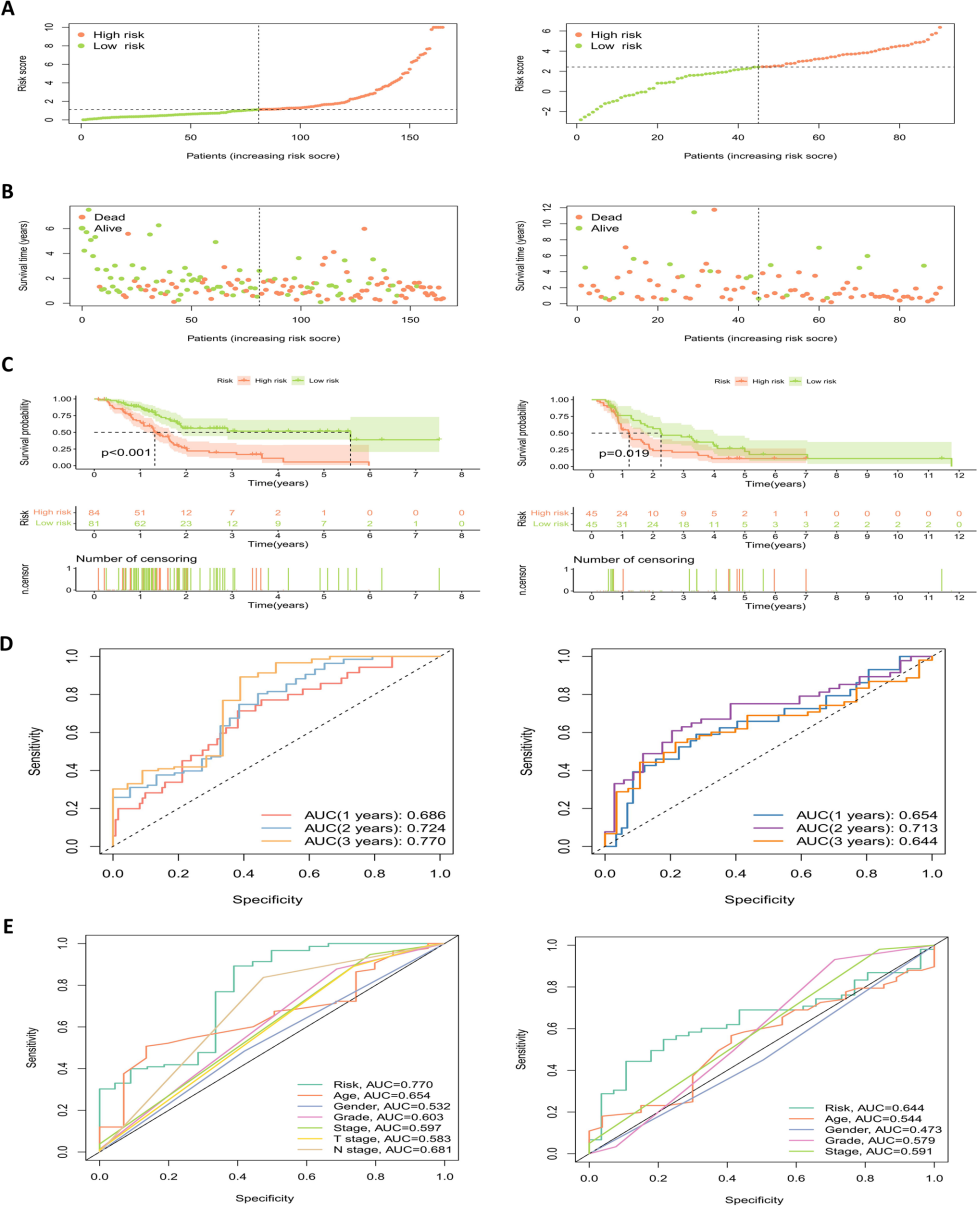
External validation of the prognostic model. **A** and **B** Distribution of risk score (**A**) and survival status (**B**) for risk subgroups in the TCGA (left) and ICGC (right) cohorts. **C** Kaplan–Meier survival curves for overall survival of patients in the TCGA (left) and ICGC (right) cohorts. **D** Temporal ROC curves for prediction of patients in TCGA (left) and ICGC (right) cohorts. **E** ROC curves of clinical characteristics of patients in TCGA (left) and ICGC (right) cohorts

**Fig. 8:**
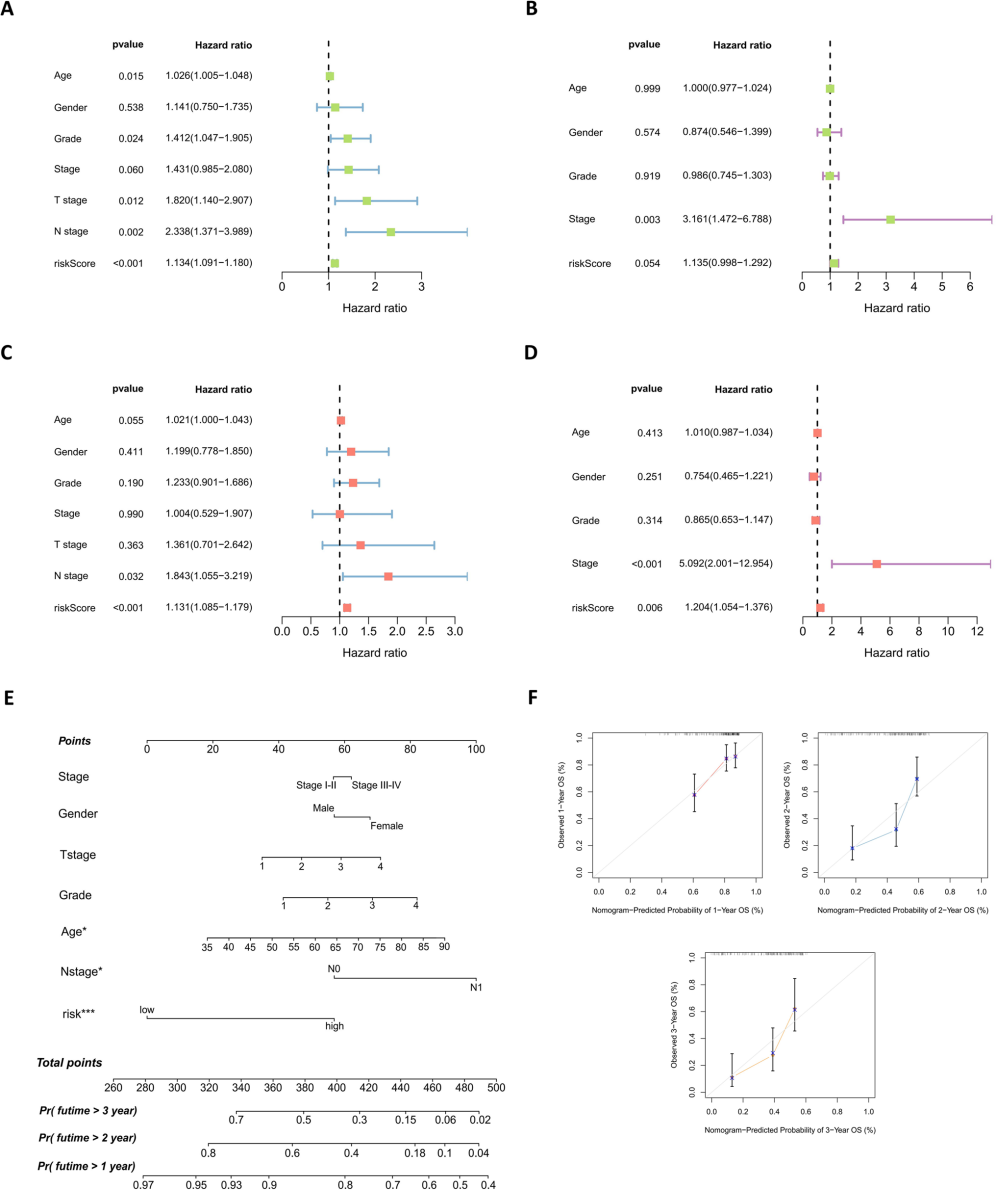
Clinical efficacy evaluation of the prognostic model. Univariate Cox regression analysis of the clinicopathological features in TCGA (**A**) and ICGC (**B**) cohorts. Forest plots showing hazard ratio and 95% confidence intervals (CI) for clinicopathological characteristics of TCGA (**C**) and ICGC (**D**) cohorts calculated by performing multivariate Cox regression analysis. **E** Nomogram of risk score and other clinical factors for predicting 1-, 2-, and 3-year overall survival in pancreatic cancer in TCGA cohort. **F** Calibration plot of the nomogram in TCGA cohort

Data from the ICGC database were applied for external validation (Table 2), comparing the survival of the model low- and high-risk groups across the TCGA database and the ICGC database (Fig. 7A–C; Supplementary Table S4, S5). In the two groups of data, the prognosis of the low-risk group was higher compared with that of the high-risk group, and the area under the ROC curve of both groups was greater than 0.65 (Fig. 7D). In addition, clinical ROC curves in both cohorts showed that the area under curve of the model risk score was the largest, which to some extent highlighted the stability of the model (Fig. 7E). Next, univariate (Fig. 8A, B) and multivariate (Fig. 8C, D) Cox regression analysis of the clinical characteristics of both cohorts showed that the risk score of the model can be used as an independent prognostic factor. Except for the risk scores that were meaningful in both sets of data, the *P* values for tumor stage in the ICGC database and N stage in the TCGA database were both less than 0.05. Finally, based on the model, a nomogram was generated for 1 to 3 years (Fig. 8E) and the expected effect verification was considerable (Fig. 8F).

**Table 2:** Clinical information for the TCGA and ICGC cohorts

Clinical feature	Type	TCGA cohort	ICGC cohort	Total
Age (years)	≤65	84 (65.12%)	45 (34.88%)	129 (51.19%)
	>65	77 (62.60%)	46 (37.40%)	123 (48.81%)
Gender	Female	74 (63.79%)	42 (36.21%)	116 (46.03%)
	Male	87 (63.97%)	49 (36.03%)	136 (53.97%)
Tumor grade	G1	25 (67.57%)	12 (32.43%)	37 (14.68%)
	G2	89 (70.63%)	37 (29.37%)	126 (50.00%)
	G3	46 (55.42%)	37 (44.58%)	83 (32.94%)
	G4	1 (16.67%)	5 (83.33%)	6 (2.38%)
Tumor stage	I	17 (73.91%)	6 (26.09%)	23 (9.13%)
	II	137 (62.56%)	82 (37.44%)	219 (86.90%)
	III	3 (60.00%)	2 (40.00%)	5 (1.98%)
	IV	4 (80.00%)	1 (20.00%)	5 (1.98%)

### Mutation status and TMB differences

**Fig. 9:**
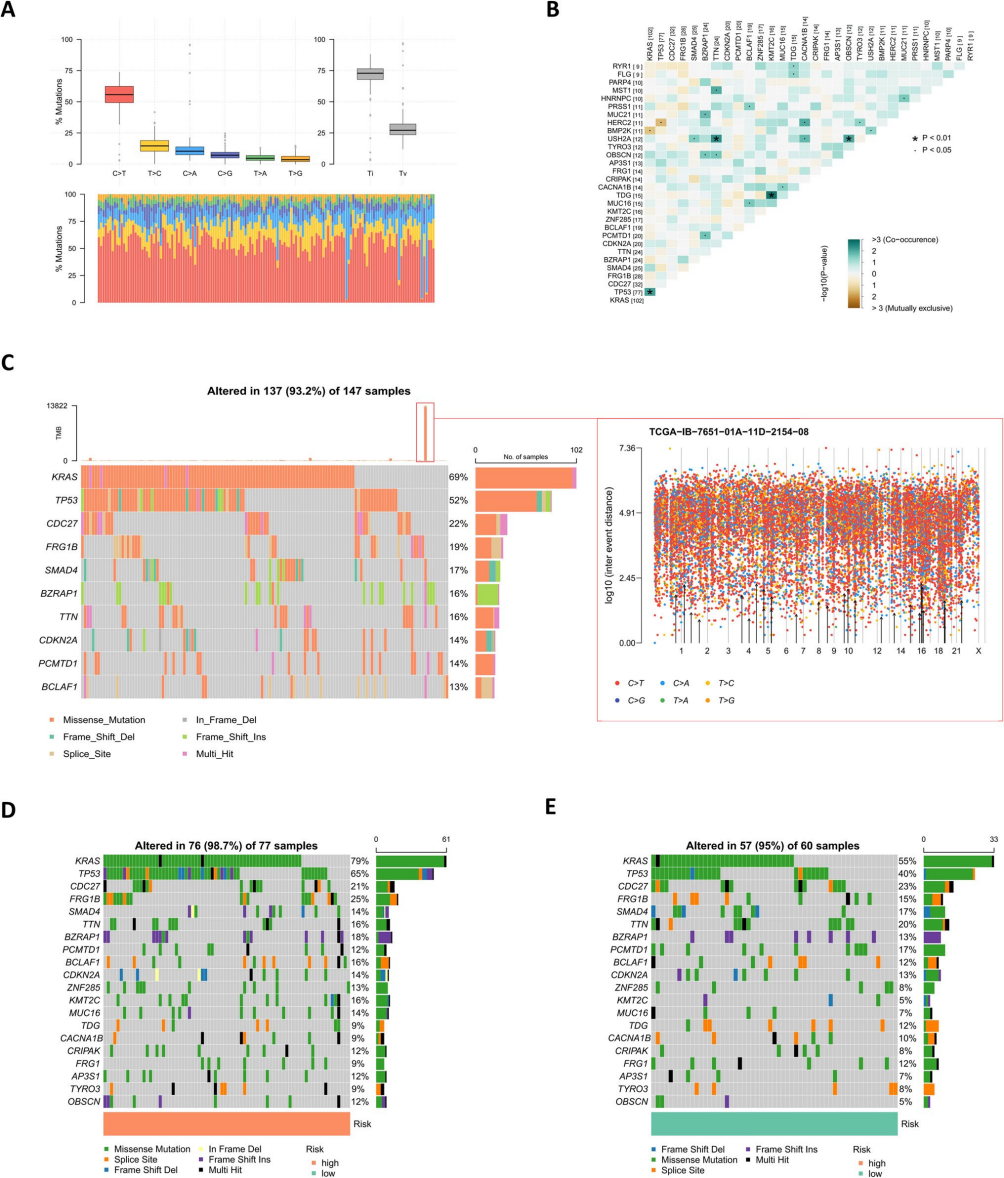
Gene mutation analysis of risk subgroups. **A** Mutational status of base sequences in the TCGA cohort. **B** Heatmap of the correlations of the top 30 mutated genes in TCGA cohort. **C** Waterfall plot of tumors in TCGA cohort (left) and mutational status of the base sequence of the TCGA-IB-7651-01A-11D-2154-08 hyper-mutations sample (right). **D** and **E** Waterfall plots of tumors with high (**D**) and low (**E**) risk scores in TCGA cohort. Individual patients are represented in each column

**Fig. 10:**
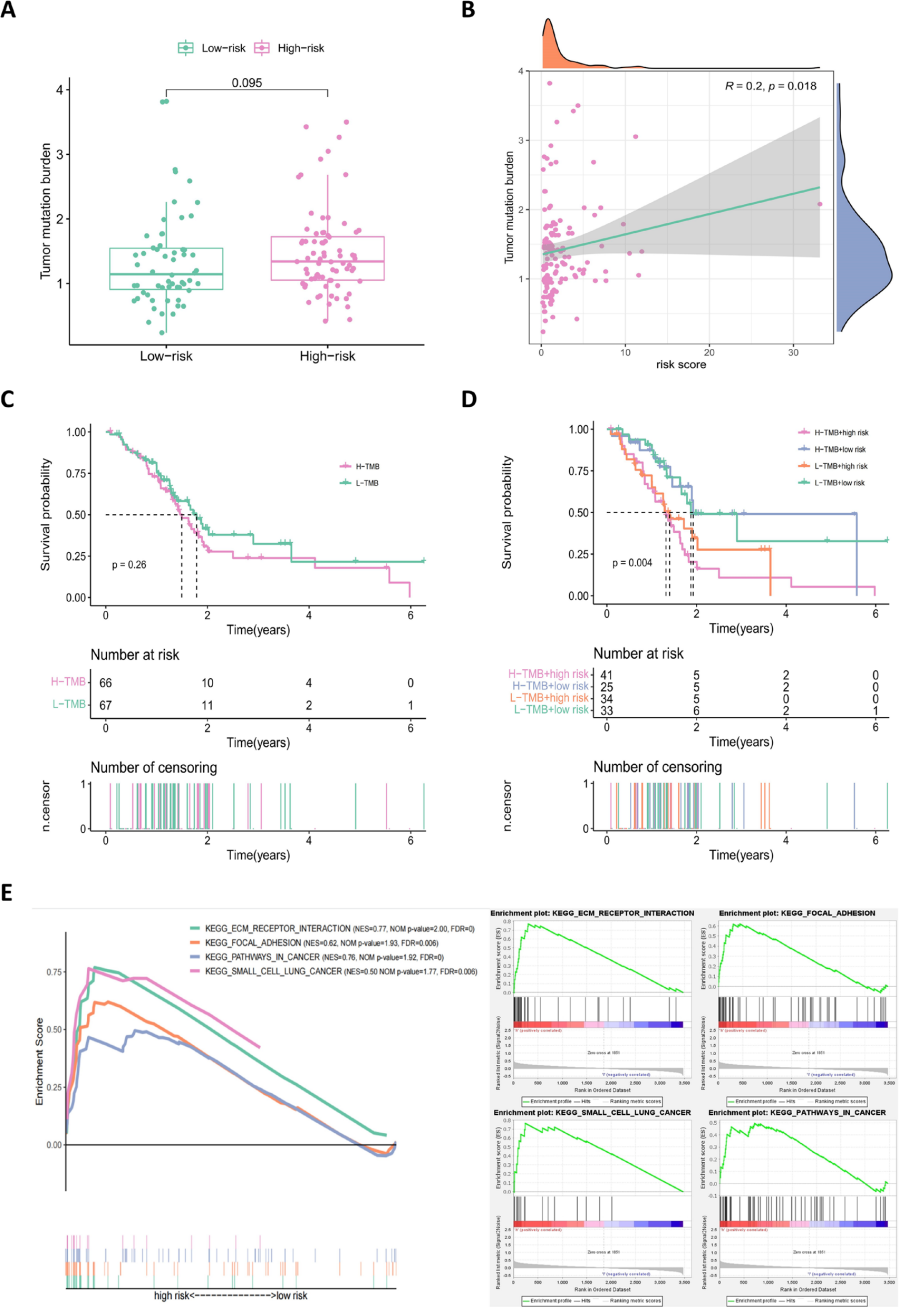
Analysis of TMB in the prognostic model. **A** Comparison of TMB between the low- and high-risk groups in the TCGA cohort. **B** Scatter plot depicting a positive correlation between risk score and mutation load. **C** Kaplan–Meier survival curves of patients in high- and low-TMB groups (H-TMB and L-TMB, respectively), grouped by the median TMB. **D** Kaplan–Meier survival curves for overall survival in four patient groups stratified by TMB and risk scores. **E** Line graphs demonstrating that GSEA-enriched KEGG pathways were significantly associated with the high-risk group in the TCGA cohort

The mutation status of the samples obtained from TCGA showed that mutual substitution between cytosine and thymine was the most common mutation, transitions occurred more frequently than transversions, and the Ti/Tv ratio was 2.57 (Fig. 9A). The correlation heatmap of the top 30 mutated genes suggested that *KRAS* and *TP53*, and *TTN* and *USH2A* were closely related (Fig. 9B). Next, waterfall plots of the samples were produced, and the top 10 mutated genes were listed, with *KRAS* and *TP53* ranking first and second, respectively (Fig. 9C; Supplementary Table S6). Based on the TMB value, hyper-mutation samples similar to TCGA-IB-7651-01A-11D-2154-08 were screened out (Fig. 9C). To enhance the objectivity of the mutation data, waterfall charts of the low- and the high-risk groups were drawn, and the high-risk group presented a significantly higher gene mutation rate (Fig. 9D, E). There was no difference in TMB value between the low- and high-risk groups following removal of the samples with hyper-mutations (Fig. 10A), but a correlation analysis between risk scores and TMB values showed a positive correlation (Fig. 10B). The survival curve indicated that the grouping of L-TMB group and H-TMB group had no effect on survival time (Fig. 10C). In TMB grouping combined with low- and high-risk groups, the grouping influenced survival, with the high-risk group having a significantly lower prognosis compared with the low-risk group (Fig. 10D). In addition, GSEA software was utilized to enrich the high-risk group, and the ECM receptor interaction pathway ranked number one (Fig. 10E).

### Differences in tumor-infiltrating immune cells

**Fig. 11:**
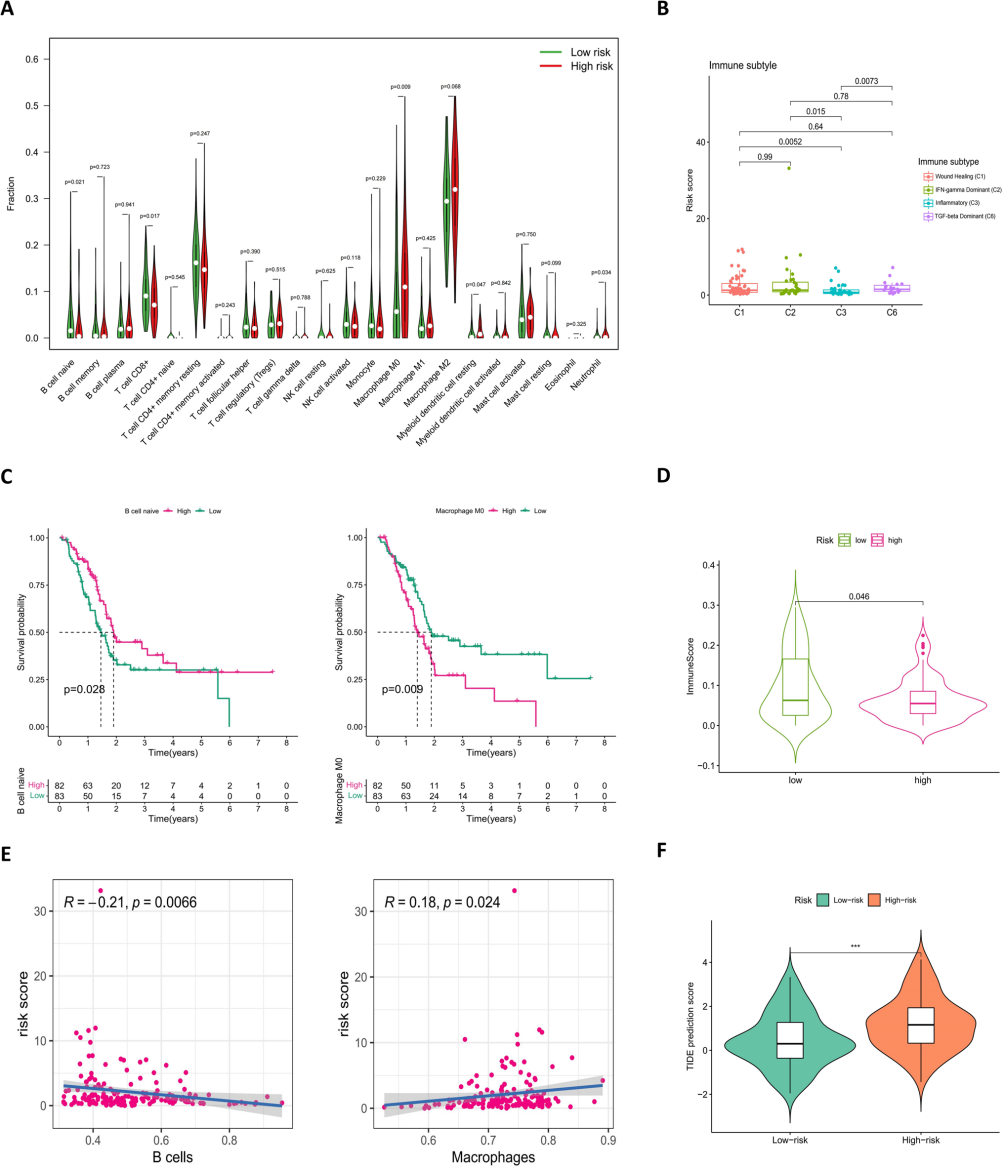
Correlation between the risk signature with tumor immune microenvironment. **A** Violin plot comparing tumor-infiltrating immune cell scores between low- and high-risk groups from the Timer2 database. **B** Boxplot showing the distribution of immune subtypes in the TCGA cohort. **C** Kaplan–Meier survival curves of naive B cell (left) and M0 macrophage (right) groups from patients in the cohort, dividing with their own median infiltration scores as a cutoff, respectively. **D** Boxplot comparing the immune scores between the high- and low-risk groups. **E** Association between risk scores and tumor-infiltrating immune cells including B cells (left) and macrophages (right). **F** Comparison of TIDE prediction scores between the low- and high-risk groups in the TCGA cohort

Five groups of immune cells including naive B cells and M0 macrophages were statistically different based on analysis of tumor-infiltrating immune cells between low- and high-risk groups (Fig. 11A; Supplementary Fig. S1). Immunophenotyping indicated that the immune types of the samples were concentrated in four types: C1, C2, C3, and C6 (Fig. 11B; Supplementary Table S7). A survival analysis revealed that naive B cell and M0 macrophage cell scores significantly affected survival (Fig. 11C). With the microenvironmental immune cell score, there were significant differences between the low- and high-risk groups (Fig. 11D), and a scatter plot indicated that risk scores were positively related to immune cell scores (Fig. 11E). In addition, TIDE scores prediction suggested that the high-risk group had a higher score and a higher possibility of immune escape compared with the low-risk group (Fig. 11F; Supplementary Table S8).

### PCA of model and potential immune drug prediction

**Fig. 12:**
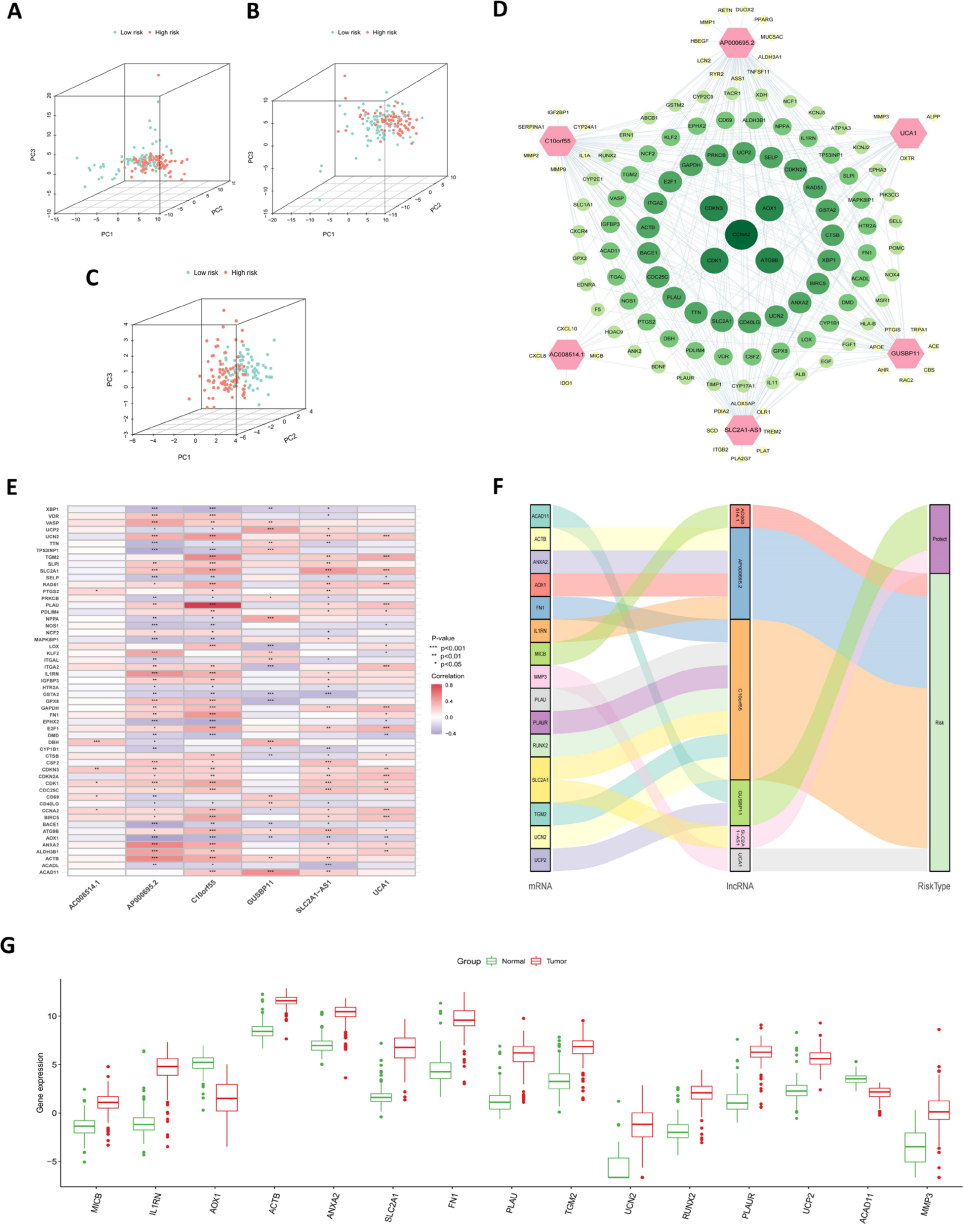
PCA and associated genes of prognostic models. **A**–**C** PCA of target genes (**A**), potential lncRNAs (**B**), and model lncRNAs (**C**) constructed separately in the TCGA cohort. **D** Network diagram of model lncRNAs and differentially expressed genes. Red hexagon represents model lncRNAs; green circle represents differentially expressed genes, with the size representing the number of related model lncRNAs (the larger the circle, the more related model lncRNAs). **E** Heatmap of correlations between model lncRNAs and differentially expressed genes. **F** Sankey diagram of differentially expressed genes significantly correlated with model lncRNAs. **G** Boxplots of differentially expressed genes between tumor and normal tissues

**Fig. 13:**
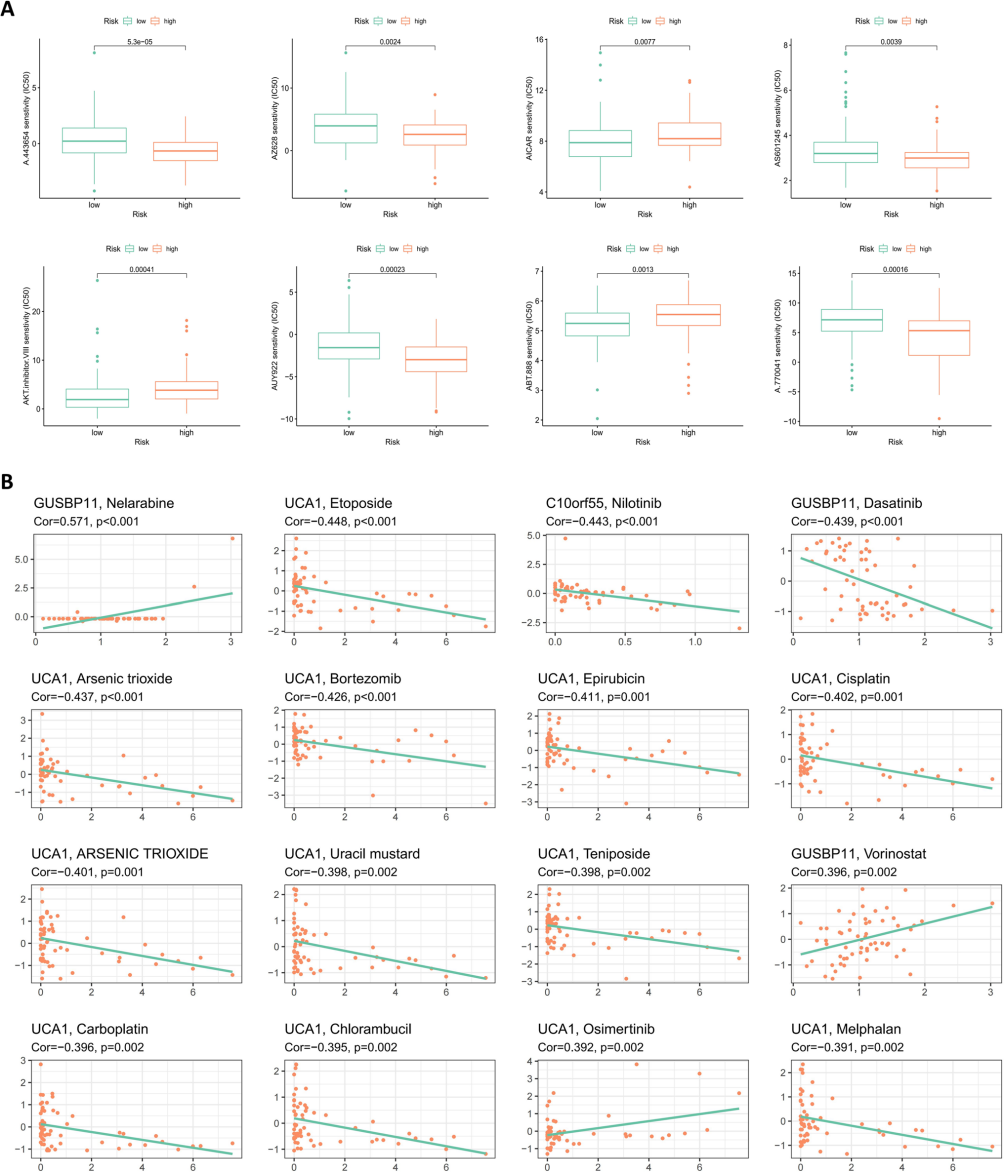
Immune-targeted drug prediction of prognostic model genes. **A** Boxplots of therapeutic drugs with different half-maximal inhibitory concentration in risk subgroups. **B** Scatter plot of model lncRNAs and immune-targeted drugs with potential efficacy

PCA constructed separately for potential target genes, potential lncRNAs, and model lncRNAs revealed differences in composition based on low- and high-risk groups (Fig. 12A–C). To further reveal the genes potentially associated with the model lncRNAs, we have mapped a network diagram, a correlation heatmap, and a Sankey diagram, respectively. The network diagram shows the association between the model lncRNAs and differentially expressed genes (Fig. 12D), and the correlation heatmap shows the differentially expressed genes associated with more than 3-model lncRNAs (Fig. 12E; Supplementary Table S9). The target genes closely related to the model lncRNAs were presented in the form of a Sankey diagram (Fig. 12F), and their expression was different between normal and tumor samples (Fig. 12G). Furthermore, the difference between the low- and high-risk groups on the half-maximal inhibitory concentration of the drug (Fig. 13A) was utilized to screen potential beneficial therapeutic drugs and possible potential drugs were predicted according to the expression of the model lncRNAs (Fig. 13B).

## Discussion

WGCNA was constructed to select green module genes related to clinical traits for Cox regression and lasso regression analysis, and a risk signature was established comprising six lncRNAs—*AC008514.1*, *AP000695.2*, *C10orf5*, *GUSBP11*, *SLC2A1-AS1*, and *UCA1*. Among them, high expression of *GUSBP11* and *SLC2A1-AS1* is beneficial to the prognosis of patients. Subsequently, based on internal and external validation, Kaplan–Meier survival analysis was performed on subgroups, which confirmed that the survival time of patients in the high-risk group was significantly shorter compared with that in the low-risk group. However, we noticed that in the Kaplan–Meier survival analysis curve of the G3-G4 subgroup of clinical characteristics, there was no statistical difference but there was still a certain trend. We speculated that this may be due to the scarcity of samples in the G4 subgroup in this analysis. In the subgroup internal and external validation, the area under the temporal ROC curve was larger than 0.66, and the risk score in the clinical characteristic ROC curve was also higher compared with other clinical indicators. Furthermore, risk score as an independent predictor was associated with the prognosis of patients with pancreatic cancer, as confirmed by Cox regression analysis. Therefore, we are confident that the risk signature identified in this study has a prognostic value in patients with pancreatic cancer. In addition, KEGG and GO analyses of genes were enriched in oxidative stress and acidic cancer metabolism-related pathways, which may be related to the characteristics of the hypoxic microenvironment of pancreatic cancer.

The mutational analysis showed an elevated Ti/Tv ratio. This may be due to the presence of many methylated cytosines in whole-exome CpG islands, and the probability of deamination of methylated cytosine to thymine is higher compared with that of other variant types (Tomkova et al., 2016). The mutation status of the TCGA cohort showed that *KRAS*, *TP53*, and *CDC27* gene mutations are the predominant mutations, and the subgroup mutation status showed that the average mutation rate of genes in the high-risk group was higher compared with that in the low-risk group, indicating that the high-risk group has a worse treatment expectation and a shorter prognosis compared with the low-risk group. To further explore the impact of mutations on the prognosis of risk subgroups, TMB was included in the mutation analysis, and this revealed that although TMB was correlated with risk scores, the difference in subgroups was not statistically significant. The H-TMB and L-TMB groups showed a certain trend of difference in the survival time of patients, but the difference failed to reach statistical significance, which may be due to the small sample size of these mutation data. The combined analysis of TMB and risk subgroups showed statistical differences, further highlighting the validity and accuracy of the prognostic model. Furthermore, four pathways—ECM receptor interaction, focal adhesion, small cell lung cancer, and pathways in cancer—were identified in the GSEA enrichment of the high-risk group. Among them, the ECM receptor interaction pathway and the focal adhesion pathway play a role in tumor cell survival, proliferation, and migration, and have been implicated in therapeutic approaches to limit tumor metastasis and promote T cell migration to tumors (Nicolas-Boluda et al., 2021; Blair et al., 2022).

Immunotherapy is an important part of the tumor treatment process. In the tumor-infiltrating immune cells of patients with pancreatic cancer, five types of immune cells, including CD8+ T cells, myeloid dendritic cells, naive B cells, M0 macrophages, and neutrophils, differed among the risk subgroups. Among these cell types, naive B cells and M0 macrophages influence patient survival. The presence of B cells is known to be associated with improved prognosis in patients with cancer (Wouters & Nelson, 2018). In addition to the ability to produce cytokines and differentiate into plasmablasts, the stability and strength of B-cell responses to T cells in cancer are altered under the influence of the tumor microenvironment (Downs-Canner et al., 2022). M0 macrophages can polarize into M1 and M2 macrophages, M1 macrophages can phagocytose cancer cells, and M2 macrophages may suppress inflammatory responses and repair tissues. However, as an anticancer therapeutic strategy for photodynamic immunotherapy, type I photosensitizers of TPA-DCR nanoparticles (NPs) can improve the immunosuppressive microenvironment under the hypoxic conditions of solid tumors by promoting the polarization of M0 and M2 macrophages to the M1 state (Yang et al., 2021). In conclusion, the difference between naive B cells and M0 macrophages in the low- and high-risk groups in the current study provides a possible immunotherapy direction for patients with pancreatic cancer. It is thought-provoking that the score of CD8+ T cells, a specific killer cell, failed to differentiate the survival time of the risk subgroup, and the immune cell infiltration data suggested that the patient’s immune cells contained many M2 macrophages. This phenomenon might be caused by immune escape following changes in the tumor microenvironment, and subsequent TIDE scores partially confirmed this theory; higher scores in the high-risk group indicated that patients in the high-risk group were more likely to evade immune strategies and less likely to benefit from immune checkpoint inhibitor therapy. Furthermore, previous studies on immune subtypes indicated that the C3 subtype is characterized by a marked type I inflammatory response, and that favorable prognosis of cancer may be due to the achievement of immune balance (Thorsson et al., 2018). Immunophenotyping in the current study revealed that patients in the low-risk group accounted for the largest proportion of the C3 subtype, which partly supported the efficacy and accuracy of the prognostic model.

Immune-targeted drugs may demonstrate therapeutic potential when tumor cells develop resistance to conventional chemotherapeutics. In the present study, PCA of the model consisting of six lncRNAs showed that the patients were significantly divided into high-risk and low-risk groups, with *GUSBP11* and *SLC2A1-AS1* recognized as protective genes in the model. High expression of *GUSBP11* in renal cancer is associated with a smaller tumor size and absence of metastasis (Jia et al., 2022), and *SLC2A1-AS1* inhibits hepatocellular carcinoma progression and glycolysis through the STAT3/FOXM1/GLUT1 axis (Shang et al., 2020). Similarly, as model risk genes, studies have indicated that *UCA1* positively regulates DLL4 expression by sponging miR-182-5p, thereby playing an oncogenic role in renal cancer pathogenesis (Wang et al., 2020). In addition, *AP000695.2* was selected as a key prognostic lncRNA to explore the prognosis of gastric adenocarcinoma (Zhang et al., 2022). In the current study, the genes closely related to the six model lncRNAs were listed and were found to be differentially expressed between normal and cancer samples; thus, they could serve as potential gene targets for regulating tumor progression. Finally, potential therapeutic drugs were screened in the present study for reference, based on half-maximal inhibitory concentration and model genes for risk subgroups.

Overall, although a prognostic risk signature model was constructed for pancreatic cancer, there are some limitations to the current study. As the study is retrospective, it is susceptible to the inherent biases of this research paradigm (Jiang et al., 2016). We tried to cite more databases as model validation, but we did not obtain proper lncRNAs information even though we retrieved the relevant information of pancreatic cancer patient matrix. This may be due to certain biases and limitations of commercial microarray databases compared to public databases such as ICGC and TCGA. However, the immunecell scores of the microenvironment and TIDE prediction scores are derived from the analysis results of multiple platforms, in a sense, this can be regarded as a data supplement validation of multiple databases. The risk signature genes have not currently been investigated in cellular experiments. However, the superior value of the risk signature has been validated in terms of survival time, clinicopathological features, tumor mutation status, tumor-infiltrating immune cells, signaling pathways, and potential small-molecule drugs, which indicates that the prognostic risk signature model is reliable. Future work will involve further exploration and validation of the risk signature with more data and larger clinical sample sizes.

## Conclusion

Using WGCNA to assess prognosis-related genes and combining lasso regression and Cox regression analysis established a new signature that may be more accurate and effective in predicting the prognosis of patients with pancreatic cancer. The signature facilitates the selection of a more appropriate and accurate immunotherapy approach for grouping treatment of patients, with potential as an independent prognostic biomarker and a predictor of immunotherapy in patients with pancreatic cancer.

## Supplementary information

ESM 1 (XLSX 258 kb)

ESM 2 (XLSX 6597 kb)

ESM 3 (XLSX 16 kb)

ESM 4 (XLSX 25 kb)

ESM 5 (XLSX 16 kb)

ESM 6 (XLSX 12 kb)

ESM 7 (DOCX 14860 kb)

ESM 8 (XLSX 12 kb)

ESM 9 (XLSX 13 kb)

ESM 10 (XLSX 21 kb)

## Data Availability

The pancreatic sample datasets were retrieved from the GTEx (https://gtexportal.org/home/), TCGA (https://dcc.icgc.org/), and ICGC (https://dcc.icgc.org/) databases.
