# Supplementary material for: Construction of a pancreatic cancer prediction model for oxidative stress-related lncRNA
Source: Funct Integr Genomics. 2023 Apr 5;23(2):118. doi: 10.1007/s10142-023-01048-6 (PMC10076407; doi:10.1007/s10142-023-01048-6)
Supplement: Supplementary file 7 — (DOCX 14860 kb) [file 10142_2023_1048_MOESM7_ESM.docx]

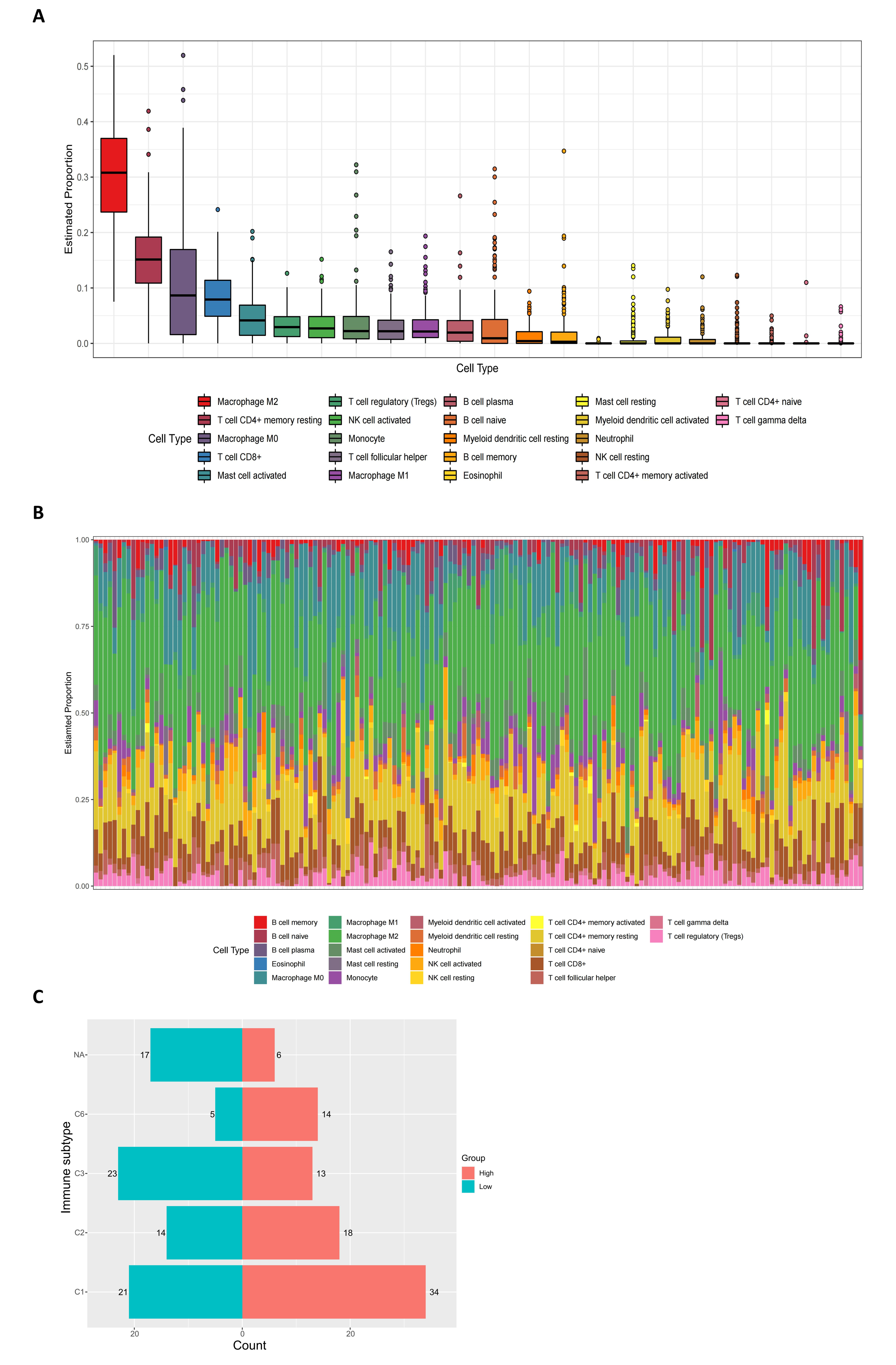


Supplementary Figure S1. (A) Boxplots showing estimated proportions of various types of immune cells in the TCGA cohort. (B) Bar chart showing the infiltration of various types of immune cells in the TCGA cohort. (C) Bar chart showing the number of immune subtype categories in risk subgroups.
